# Bridging the gap: advancing cancer cell culture to reveal key metabolic targets

**DOI:** 10.3389/fonc.2024.1480613

**Published:** 2024-09-17

**Authors:** Marjolein M. G. Kes, Celia R. Berkers, Jarno Drost

**Affiliations:** ^1^ Princess Máxima Center for Pediatric Oncology, Oncode Institute, Utrecht, Netherlands; ^2^ Division Cell Biology, Metabolism & Cancer, Department Biomolecular Health Sciences, Faculty of Veterinary Medicine, Utrecht University, Utrecht, Netherlands

**Keywords:** cancer metabolism, cell culture conditions, organoids, tumor microenvironment, physiologic media, oxygen, pH

## Abstract

Metabolic rewiring is a defining characteristic of cancer cells, driving their ability to proliferate. Leveraging these metabolic vulnerabilities for therapeutic purposes has a long and impactful history, with the advent of antimetabolites marking a significant breakthrough in cancer treatment. Despite this, only a few *in vitro* metabolic discoveries have been successfully translated into effective clinical therapies. This limited translatability is partially due to the use of simplistic *in vitro* models that do not accurately reflect the tumor microenvironment. This Review examines the effects of current cell culture practices on cancer cell metabolism and highlights recent advancements in establishing more physiologically relevant *in vitro* culture conditions and technologies, such as organoids. Applying these improvements may bridge the gap between *in vitro* and *in vivo* findings, facilitating the development of innovative metabolic therapies for cancer.

## 1 Introduction

Cancer cells can autonomously rewire their metabolic pathway activity to meet their increased bioenergetic, biosynthetic, and redox needs (1). These reprogramming activities are observed ubiquitously across many cancer types and are therefore considered a hallmark of cancer (2, 3). Established in the 1920s with Otto Warburg’s pioneering work on aerobic glycolysis (4), the study of cancer metabolism represents one of the oldest areas of research in cancer biology (1).

The concept of exploiting metabolic vulnerabilities as a cancer therapy is longstanding (5), with the first antimetabolite therapy dating back to 1948 (6). At that time, Farber and colleagues demonstrated that aminopterin, a folate analogue blocking *de novo* nucleotide biosynthesis, could halt tumor progression in children with acute lymphoblastic leukemia (ALL) (6). Since then, several other antimetabolites including methotrexate, 6-mercaptopurine and 5-fluorouracil (5-FU) have found their way into the clinic and are now being extensively used in various cancer treatment regimens (7–9).

Despite these advancements, only a limited number of identified metabolic vulnerabilities have been successfully translated into effective (targeted) therapies to date. This is, at least in part, due to the use of reductionist *in vitro* models in preclinical studies that fail to recapitulate the complex microenvironment that defines the heterogeneous metabolic landscape of human tumors (10, 11). Furthermore, it has become increasingly appreciated that the artificial environment of cell culture systems dictates the metabolic state of cancer cells and that minor adjustments in cancer modeling (i.e., cell culture architecture and microenvironmental interactions), biochemical (i.e., nutrients, cell culture media) and physicochemical (i.e., oxygen levels, pH) factors could easily alter metabolic pathway activity, thereby influencing metabolic readouts (12, 13).

In this Review, we discuss the impact of current cancer models, biochemical and physiochemical conditions in standard cell culture practices on cancer cell metabolism. Furthermore, recent efforts to improve the modeling capacity of *in vitro* systems to better recapitulate physiologic conditions are discussed, including their strengths and current limitations.

## 2 Modeling of tumor tissue architecture and microenvironmental interactions enhances the metabolic fidelity of *in vitro* cancer models

Conventional cancer cell metabolism studies have predominantly been conducted using two-dimensional (2D) cell cultures. However, cells grown as a monolayer do not accurately replicate the three-dimensional (3D) growth dynamics of a tumor (14). Moreover, gaining comprehensive insights into cancer metabolism requires models that facilitate the study of intra- and intercellular communication and tumor-microenvironment (TME) interactions. 3D cell culture platforms, including tumor spheroids and organoids, replicate many of the pathophysiological features of solid tumors, such as cell-cell contacts as well as pH, oxygen and nutrient gradients (15). Such models are increasingly favored to study tumor biological processes *in vitro (* 16), but also exhibit several drawbacks. Currently used 3D cultures lack many components of the TME that shape the unique metabolic landscape of patient tumors, such as infiltrating stromal and immune cells, vasculature and the tumor interstitial fluid (17). Below, we discuss recent efforts in optimizing cell culture dimensionality (e.g., 3D cultures) and complexity (e.g., co-cultures, tissue explants, microfluidics) that have led to *in vitro* tumor models that better recapitulate the metabolic landscape of tumors.

### 2.1 Cell culture architecture: 2D versus 3D

3D cell culture models are a promising tool to mitigate the gap between 2D culture systems and cancer tissues to study the metabolic complexity of cancer (18). Various studies tried to assess how the metabolic profile of 3D models compares to that of 2D cultures and are reviewed in **Tables 1**, **2**. Most of these studies report an increase in the glycolytic- (19, 20, 42, 43) and oxidative capacities (20, 21, 42) of 3D cultures. Yet, several studies report the opposite, with diminished glycolysis (21) or oxidative metabolism (19, 43) present in 3D models. Moreover, 3D models showed a higher maintenance of ATP production (20, 42, 44, 45), redox balance (42, 44), and biomass synthesis, including nucleotides (42, 44), amino acids (21, 42, 44), lipids (22, 23, 42–45) and NADPH (42, 44). Nevertheless, reductions in amino acid (43) and especially *de novo* nucleotide synthesis (24, 43, 45) were also reported for 3D models compared to their 2D counterparts. Because these comparative studies were conducted in models of various cancer types, it is believed that these contradictory results can be partially attributed to the metabolic variation across tumor entities. Although a considerable number of studies have addressed the metabolic differences between 2D and 3D cultures, fewer studies make the comparison between these *in vitro* systems and the metabolic profiles found in primary tumor tissues. Nevertheless, studies that do investigate this indicate a closer resemblance of 3D models to cancer tissues compared to either the traditional 2D cultures (44) or normal, non-cancerous tissue (45).

**Table 1:** Cited literature on more common cancer types that review the impact of cell culture conditions on cancer cell metabolism *in vitro*.

Review of cited literature on common cancer types		
Authors	Cancer type	Metabolic impact of cell culture conditions
CELL CULTURE PARAMETER		
Comparison of 2D cultures versus 3D cultures		
Rodríguez-Enríquez et al., 2008 (19)	Cervical cancer	Increased glycolytic flux and decreased oxidative phosphorylation potential in 3D HeLa cultures.
Tidwell et al., 2022 (20)	Colorectal cancer	Increased glycolytic activity, ATP-linked respiration and non-aerobic ATP production in 3D colorectal cancer and PDAC cell cultures.
Sato et al., 2016 (21)	Ovarian and cervical cancer	Decreased lactate production (glycolysis) and increased amino acids (serine, aspartate, glutamate, glutamine), citrate (TCA cycle) activity in ovarian and cervical cancer 3D cultures.
Tobias & Hummon, 2022 (22)	Colon cancer	Increased sphingolipid, acylcarnitine, polyunsaturated fatty acid (PUFAs), and lipid subclasses associated with lipid droplets (triacylglycerol) production in 3D colon cancer cultures.
Vidavski et al., 2019 (23)	Breast cancer	When moving from 2D to 3D breast cancer cultures, total lipid amount decreased, while the neutral glycerolipids, ratio of acylglycerols to membrane lipids and formation of large lipid droplets increased.
Fan et al., 2018 (24)	Lung cancer	Similar ^13^C_6_-glucose incorporation into glycolytic, TCA, PPP, and nucleotide biosynthesis metabolites in 2D and 3D lung cancer cultures. Reduced *de novo* pyrimidine and sugar nucleotide synthesis in 3D cultures. Selenite treatment induced lesser perturbation of metabolic pathways in 3D cultures.
Russell et al., 2017 (25)	Colon and lung cancer	Differential metabolism in 2D and 3D colon- and lung cancer cell models result in different responses to chemotherapeutic drugs, with 2D models being more sensitive than 3D models.
Comparison of standard culture media vs physiologic culture media		
Cantor et al., 2017 (26)	Various cancer types	HPLM had profound effects on abundance of amino acid, lipid, and nucleotide metabolism, redox state and glucose utilization of cancer cells. Presence of uric acid in HPLM lead to inhibition of *de novo* pyrimidine synthesis enzyme UMPS, reducing sensitivity of cancer cell lines to 5-FU.
Vande Voorde et al., 2019 (27)	Breast cancer	Reduced intracellular pyruvate levels, reduced uptake of glutamine and proportional changes in uptake/release of other amino acids of triple-negative breast cancer cells cultured in Plasmax. Better recapitulation of the metabolic signature of orthotopic xenograft models by cells cultured in Plasmax.
Golikov et al., 2021 (28)	Cervical and lung cancer	Higher basal and maximum respiration levels with almost no effect on glycolysis for cervical and lung carcinoma cells cultured in Plasmax.
Moradi et al., 2021 (29)	Various cancer types	Increased oxidative and decreased glycolytic metabolism in cancer cells cultured n Plasmax.
Comparison of normoxic O_2_ levels versus physioxic or hypoxic O_2_ levels		
Moradi et al., 2021 (29)	Various cancer types	Increased mitochondrial metabolism in three out of the four human cancer cell lines at physioxic (5%) compared to normoxic (18%) O_2_ conditions.
Timpano et al., 2019 (30)	Breast cancer	Significantly increased glycolysis at 1% O_2_ compared to normoxic breast cancer cells. Decreased mitochondrial activity at ≥12% O_2_ compared to physioxic breast cancer cells.
Frezza et al., 2011 (31)	Colon cancer	Compared to normoxia (21% O_2_), there was increased glycolysis, protein- and lipid catabolism at hypoxia (1% O_2_) in colon cancer cells. Retained mitochondrial-dependent oxygen consumption under hypoxia, but at significantly lower rates than normoxic cells.
Tsai et al., 2013 (32)	Breast cancer	Increased lactate, pyruvate glutamine, valine, leucine, methionine and phenylalanine metabolite levels in breast cancer cells at hypoxia (0.5% O_2_) compared to normoxia (21% O_2_). Decreased myo-inositol, formate, tyrosine, creatine, glutamate, proline, glycine, alanine and acetate levels at hypoxia.
Yang et al., 2018 (33)	Breast cancer	Increased glycolysis and decreased TCA cycle activity in breast cancer cells at hypoxia. Hypoxia decreased the flux of glucose and increased the flux of glutamine into the TCA cycle.
Martín-Bernabé et al., 2021 (34)	Lung cancer	Increased lactate production and decreased glutamine uptake in lung cancer cells at hypoxia.
Comparison of neutral pH versus acidic pH		
Chen et al., 2008 (35)	Colon and cervical cancer	Decreased glucose consumption and glycolytic metabolism in colon- and cervical cancer cells cultured at acidic pH.
Peppicelli et al., 2016 (36)	Melanoma	Decreased lactate production and increased oxidative metabolism in melanoma cells cultured at pH 6.7 compared to pH 7.4. The acidosis-induced EMT phenotype in melanoma cells could be prevented by the mitochondrial complex I inhibitor Metformin.
Corbet et al., 2016 (37)	Various cancer types	Decreased use of glucose, leading to a reduced production of acetyl-CoA by cancer cells cultured at pH 6.5 compared to pH 7.4. Concomitant use of fatty acid oxidation (FAO) and synthesis (FAS) under acidosis through downregulation of ACC2.
LaMonte et al., 2013 (38)	Breast cancer	Decreased glycolysis, lactate and glutathione production, and increased glutaminolysis, fatty acid β-oxidation, pentose phosphate pathway activity, and NADPH production of breast cancer cells cultured at pH 6.7 compared to pH 7.4.
Corbet et al., 2014 (39)	Various cancer types	Decreased glycolysis, increased reductive glutamine metabolism and glutamine-fueled oxidative phosphorylation in cancer cells cultured at pH 6.5 compared to pH 7.4. *In vivo*, glutaminase inhibitor BPTES significantly reduced growth of tumors comprised of cells pre-adapted to pH 6.5 compared to tumors from cells pre-adapted to pH 7.4.
Prado-Garcia et al., 2020 (40)	Lung cancer	Decreased lactate production in both A-549 and A-427 lung cancer cells at pH 6.2 compared to pH 7.2. Decreased glucose consumption in A-549 cells but not in A-427 cells at pH 6.2. Oxidative metabolism increased in A-427, but decreased in A-549 cells at pH 6.2.
Rolver et al., 2022 (41)	Various cancer types	Increased oxidative metabolism, fatty acid uptake, fatty acid oxidation (FAO) and lipid accumulation in cancer cells cultured at pH 6.5 compared to pH 7.6.

**Table 2:** Cited literature on more rare cancer types that review the impact of cell culture conditions on cancer cell metabolism *in vitro*.

Review of cited literature on rare cancer types		
Authors	Cancer type	Metabolic impact of cell culture conditions
CELL CULTURE PARAMETER		
Comparison of 2D cultures versus 3D cultures		
Ikari et al., 2021 (42)	Bladder cancer	Significantly lower levels of most metabolites, including glycolytic- and TCA cycle intermediates in 2D prostate- and bladder cancer cultures. Higher maintenance of ATP production, biomass (nucleotides, amino acids, lipids and NADPH) synthesis, and redox balance in 3D cultures.
Wen et al., 2023 (43)	Glioma	Decreased nucleotide, amino acid and glutathione metabolism in 3D glioma cultures. Fluxomics analysis indicates increased glycolysis and *de novo* lipid biosynthesis activity, and decreased TCA cycle and *de novo* purine biosynthesis activity in 3D glioma cultures.
Tidwell et al., 2022 (20)	Pancreatic cancer	Increased glycolytic activity, ATP-linked respiration and non-aerobic ATP production in 3D PDAC cell cultures.
Murakami et al., 2020 (44)	Tongue cancer	Significantly lower levels of most metabolites and loss of cancer cell line-specific metabolic profiles in tongue cancer 2D cultures. More active ATP production, biomass synthesis, and maintenance of redox balance in 3D cultures, closely resembling the metabolic activity in xenografts.
Zang et al., 2021 (45)	Esophageal cancer	Similar metabolite levels detected in 3D esophageal cancer cultures and cancer tissues compared to normal tissues. Abnormal glutamine metabolism, TCA cycle deregulation, increased energy metabolism, decreased inosine levels, and upregulation of most lipids in 3D cultures and cancer tissues compared to normal tissues.
Fan et al., 2018 (24)	Pancreatic cancer	Similar ^13^C_6_-glucose incorporation into glycolytic, TCA, PPP, and nucleotide biosynthesis metabolites in 2D and 3D pancreatic cancer cultures. Reduced *de novo* pyrimidine and sugar nucleotide synthesis in 3D cultures. Selenite treatment induced lesser perturbation of metabolic pathways in 3D cultures.
Comparison of standard culture media versus physiologic culture media		
Golikov et al., 2021 (28)	Hepatocellular cancer	Higher basal and maximum respiration levels with almost no effect on glycolysis for hepatocellular carcinoma cells cultured in Plasmax.
Saab et al., 2023 (46)	Pancreatic cancer	PDAC cells cultured in TIFM adopt a cellular state closer to tumors than standard PDAC cultures. Culturing in physiological nutrient conditions identified *de novo* arginine synthesis in PDAC as a true metabolic feature.
Khadka et al., 2021 (47)	Glioma	Glutaminolysis, but not glycolysis, is reduced in Plasmax-cultured ENO1-deleted glioma cells corresponding to the absence of *in vivo* efficacy of glutaminolysis inhibitor CB-839. In standard DMEM medium, cells with and without ENO1 deletion were equally sensitive to CB-839 treatment.
Comparison of normoxic O_2_ levels versus physioxic or hypoxic O_2_ levels		
Blandin et al., 2019 (48)	Pediatric high-grade glioma (pHGG)	Compared to normoxic conditions (21% O_2_), metabolism was significantly closer to the relapsed pHGGs and significantly different from the tumor at diagnosis under hypoxia (1% O_2_),. Decreased glucose uptake and lactate production and increased ROS, lipolysis, serinolysis, and glutaminolysis at hypoxia as well as in relapsed pHGGs.
Gunda et al., 2018 (49)	Pancreatic cancer	Increased glycolysis and an overall decrease in TCA cycle metabolites in PDAC cells under hypoxia (1% O_2_) compared to normoxia (21% O_2_)
Kucharzewska et al., 2015 (50)	Glioblastoma	Increased levels of glucose, glycolysis- and PPP intermediates, lactate production and protein catabolism in glioblastoma cells at hypoxia (1% O_2_) compared to normoxia (21% O_2_). Decreased TCA cycle intermediates and nucleotides at hypoxia.
Al-Mutawa et al., 2018 (51)	Neuroblastoma	High levels of glycolytic end-product lactate were triggered by hypoxia (1% O_2_) *in vitro*, but not by hypoxia pre-conditioned neuroblastoma tumors. The effects of hypoxia *in vitro* neuroblastoma cells did not compare with *in vivo* tumors.
Kumano et al., 2024 (52)	Pancreatic cancer	Hypoxia (1% O_2_) generated PDAC organoids with a different morphology, increased EMT-related protein expression and a higher 5-FU resistance compared to cells cultured at normoxia (20% O_2_).
Comparison of neutral pH versus acidic pH		
Hu et al., 2019 (53)	Glioma	Increased mitochondrial metabolism in stem cell-like glioma cells, but not in differentiated glioma cells cultured at pH 6.8 compared to pH 7.4.
Abrego et al., 2017 (54)	Pancreatic cancer	Decreased glucose uptake, glycolytic metabolism and glutathione levels, and increased oxidative- and anaplerotic glutamine metabolism in PDAC cells cultured in low pH 7.0 compared to pH 7.4.
Chano et al., 2016 (55)	Osteosarcoma	Decreased glycolysis and lactate production, and increased oxidative metabolism, TCA- and urea cycle, pentose phosphate pathway activity and amino acid catabolism in osteosarcoma cells cultured at pH 6.5 compared to pH 7.4. Higher sensitivity to HDAC inhibitors at pH 6.5.
Xu et al., 2021 (56)	Glioma	Increased TCA cycle flux, pentose phosphate pathway activity, *de novo* purine synthesis and glutathione levels in glioma stem cells cultured at pH 6.8 compared to pH 7.4.

Given the metabolic differences identified between 2D and 3D culture conditions, it is not surprising that these models show altered sensitivities to commonly used therapeutic agents. Several findings highlight the importance of considering 3D over or next to 2D models in pre-clinical studies evaluating cancer metabolism and responses to anti-cancer drugs, with numerous studies indicating that 3D models exhibit greater resistance to chemotherapeutics compared to 2D models (24, 25, 57–59), thereby more accurately mimicking drug responses observed *in vivo*.

A burgeoning number of studies combines 3D organoid models such as patient-derived tumor organoids (PDTO) with metabolomics and stable-isotope tracing approaches to study cancer cell metabolism (60–64). Several reports have demonstrated that this approach could facilitate the assessment of metabolic responses to treatment and aid in the development of novel metabolic treatment strategies. For instance, Neef and colleagues (63) observed dose-dependent alterations in the metabolic profiles of patient-derived colorectal cancer (CRC) organoids subjected to 5-FU treatment. Importantly, the metabolites that exhibited significant changes were primarily associated with purine and pyrimidine metabolism, consistent with the known mechanism of action of 5-FU (63). Furthermore, Ludikhuize et al. (64) investigated the 5-FU response in PDTO models mimicking the different CRC stages. They found that 5-FU induces DNA damage and cell death in p53-deficient CRC organoids due to pyrimidine imbalance, with enhanced toxicity observed in KRAS^G12D^ glycolytic CRC organoids when targeting the Warburg effect (64). Together, these studies illustrate the valuable role of 3D organoids in monitoring drug-induced metabolic changes and identifying tumor-specific metabolic sensitivities *in vitro*.

### 2.2 Tumor microenvironment interactions

The advent of co-culture systems has made it possible to incorporate multiple cell types to study metabolic cell-cell communication. These *in vitro* co-culture techniques have provided fundamental insights into the metabolic crosstalk between tumor cells and stromal cells, encompassing adipocytes (65–68), endothelial cells (69, 70) and fibroblasts (71, 72), as well as immune cells such as macrophages (73–75) and T lymphocytes (76–78). However, such systems still lack the cellular diversity as well as the matrix and vascular compartments found within the TME. To address this issue, several next-generation culture platforms have been developed. For example, several groups have set out to establish patient-derived explants (PDEs) to investigate tumor cell metabolism. PDEs are generated by directly culturing fresh, non-dissociated tumor tissue slices *in vitro*, thereby preserving native tissue architecture, TME, cell-cell interactions and metabolic crosstalk of the *in vivo* situation (79). In the past, PDEs have been used for metabolic studies (80, 81), but due to their relatively short-term viability and the lack of consistent PDE culturing methodologies their use remains limited (16).

In addition to PDEs, cancer-on-chip (CoC) platforms are emerging as advanced 3D approaches. A CoC is a micro-fluidic-based device that usually hosts multiple cell types in a more *in vivo-*like microenvironment where mechanical stimuli, flow, and rate of chemical release can be controlled (82, 83). Sensors can also be integrated to perform real-time monitoring of physicochemical cues, such as pH and O_2_ levels (84). Recently, Dornhof et al. (85) integrated biosensors to measure oxygen, lactate, and glucose into a microfluidic CoC platform, allowing precise and reproducible on-chip multi-analyte metabolite monitoring in real-time. Even more advanced is the work of Kalfe et al. (86), who embedded a microfluidic tube containing tumor spheroids directly into a miniaturized NMR metabolomics detector, allowing them to monitor 23 metabolites. Moreover, Chen et al. (87) developed a CoC integrated with electrospray ionization mass spectrometry, enabling the simultaneous measurement of drug-induced apoptosis and metabolites with high stability, sensitivity and repeatability. Indeed, microfluidic systems have been demonstrated to be able to mimic the *in vivo* tumor conditions better than traditional 2D systems (88). Especially with the integration of primary, PDTO cultures, a superior reproduction of the *in vivo* conditions could be obtained. Still, one important limitation of CoC systems is their simplicity, as these devices currently only incorporate the essential components (89).

## 3 Culture medium composition has a profound impact on cancer cell metabolism

Metabolic pathway activity is dynamically regulated in a context-dependent manner to balance the anabolic and catabolic needs within a cell. Cancer cells, which frequently encounter nutrient-poor, acidic microenvironments with restricted oxygen availability, must undergo metabolic reprogramming to adapt to and thrive under these nutritional fluctuations (90, 91). Modeling cancer cells under variable lactate and nutrient concentrations that mimic the cancer microenvironment may therefore enhance our understanding of cancer metabolism *in vivo*.

Recognition of the impact of cell culture medium composition on the transcriptomic, epigenetic and metabolic profiles of cells has grown considerably over the past years (13, 92, 93). Still, much of our current knowledge on cancer metabolism predominantly stems from studies using cells cultured in standard, nutrient-rich media. Frequently, these standard media include a largely undefined serum component (e.g., fetal bovine serum (FBS)) in conjunction with one of the several defined basal media (e.g., Minimal Essential Medium (MEM), Dulbecco’s Modified Eagle Medium (DMEM), and RPMI 1640). Such standard media were originally designed to promote the proliferation of specific cell types without the need for constant refeeding, rather than to accurately mimic the *in vivo* metabolic environment (12).

Media formulations mimicking the nutrient concentrations in plasma or the direct microenvironment can improve the biological relevance of *in vitro* cancer modeling and aid in addressing the metabolic discrepancies between *in vivo* and *in vitro* systems. In 2017 and 2019, two physiological media that more accurately represent the metabolic profile of human plasma were formulated, termed human plasma-like medium (HPLM) (26) and Plasmax (27). Multiple studies that used these media formulations (see **Tables 1**, **2**) have indicated a decreased use of glucose and reduced glycolytic activity of cancer cells cultured in physiologic medium, while the use of oxidative metabolism was shown to be increased (26, 28, 29). Profound effects on amino acid, lipid, and nucleotide metabolism have also been reported (26). Furthermore, several studies have demonstrated that culturing of cancer cells in physiological media results in metabolic profiles that more closely resemble the metabolic state of tumors (27, 46), and could lead to a better assessment of the effectiveness of antitumor drugs *in vivo* (26, 47). For example, Vande Voorde and colleagues (27) compared the metabolic profiles of CAL-120 breast cancer cells cultured in DMEM-F12 and Plasmax, both as 2D monolayers and 3D spheroids, with CAL-120-derived mammary tumors. They found that 3D spheroids cultured in Plasmax had a metabolic profile closest to that of the tumors, suggesting that 3D culture in a physiological medium better approximates the tumor’s metabolic phenotype (27). In addition, Cantor et al. (26) showed that the physiologic uric acid levels present in HPLM directly inhibit uridine monophosphate synthase (UMPS), thereby reducing the sensitivity of cancer cells to antimetabolite 5-fluorouracil.

Tumor cells are not directly exposed to nutrients in circulating plasma, but rather to nutrients present in the extracellular fluid that perfuses the tissue, so-called tumor interstitial fluid (TIF) (94). By extracting both plasma and TIF from murine lung- and pancreatic adenocarcinoma (PDAC) models, Sullivan et al. (95) revealed that the nutrients available in TIF differ from those present in plasma. Building on these findings, TIF medium (TIFM), containing nutrient levels representative of the PDAC microenvironment, was developed by the same group (46). PDAC cells cultured in TIFM more closely resembled the metabolic state of PDAC tumors compared to standard cell culture models. In addition, these TIFM-cultured PDAC models revealed high *de novo* arginine synthesis activity to be a specific metabolic feature of PDAC tumors (46). Nevertheless, it remains to be determined whether the nutrient concentrations observed in murine TIF are comparable to those in human tumors.

While these advancements in media formulation enhance the metabolic fidelity of cell culture models, physiological media are susceptible to rapid nutrient depletion (96), indicating the necessity of daily media replacement in such cultures. However, the daily renewal of nutrients and growth factors could lead to cyclic metabolic activation of cells and therefore affect experimental readouts. Instead, continuous perfusion of cells with physiologic media using a fluidic system, for example, could at least partially solve this (10).

## 4 Physicochemical culture properties influence cancer cell metabolism

Both *in vitro* and in the human body, the physicochemical environment (i.e., temperature, pH, O_2_ and CO_2_ levels) is tightly controlled, but not necessarily the same. Under normal physiological conditions, the pH of blood and tissues is tightly regulated to be at pH 7.4 (97). Similarly, a stable pH range of 7.2-7.4 is often maintained in cell culture media by the addition of buffers, such as sodium bicarbonate (NaHCO_3_) or HEPES (98). In contrast, most conventional cell culture incubators do not regulate O_2_ levels, resulting in atmospheric O_2_ concentrations around 18-21% (99), while much lower oxygen levels are found in human tissues, ranging from 3-7.4% (physioxia) (100).

**Figure 1:**
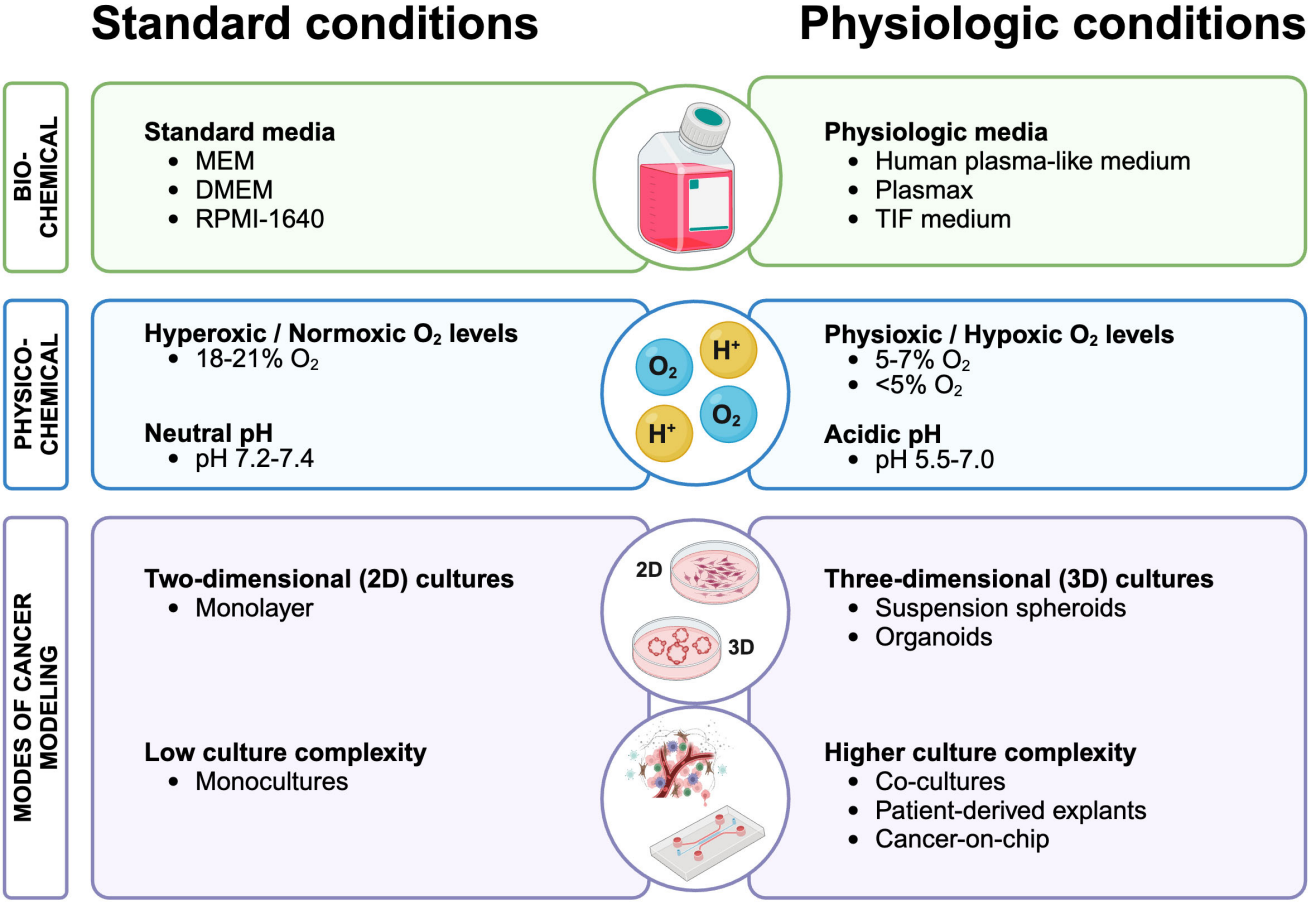
Revising cancer cell culture conditions: comparing standard practices with physiologically relevant conditions for enhanced cancer metabolism modeling. Standard cell culture practices (left) that could be improved to more accurately reflect human physiology (right) to enhance our understanding of metabolic processes in cancer. Created with BioRender.com.

In the microenvironment of human tumors, pH and oxygen levels are subjected to further change. In solid tumors, the microenvironment is frequently acidified due to elevated levels of acidic metabolites, such as lactate, and the secretion of protons (H^+^) through specific pumps and transporters (101). The latter processes result in a local extracellular pH ranging from 5.5 to 7.0 (102, 103). Extracellular acidosis is therefore considered a hallmark of most human tumors, strongly linked to malignancy, aggressiveness (36) and/or stemness (53) of tumors. Oxygenation in tumors frequently drops to 0.3-4.2% oxygen (hypoxia) (100). Yet, most *in vitro* cancer studies have been performed under hyperoxic conditions, with 18–21% O_2_ now frequently referred to as ‘normoxia’ in literature (104). Thus, adjusting O_2_ levels and pH to more physiologically relevant conditions, as reviewed below (see also **Figure 1** and **Tables 1**, **2**), can influence cancer cell metabolism *in vitro* to provide a more accurate representation of the *in vivo* tumor milieu.

### 4.1 Oxygen levels: normoxia versus physioxia and hypoxia

The supraphysiological O_2_ levels used in cell culture systems greatly impact cancer cell metabolism, and could confound the metabolic findings between *in vitro* and *in vivo* settings (99). These findings could have important implications when studying various cancer drugs that target energy metabolism. Several studies report higher mitochondrial activity at physioxia than at normoxia (29, 30, 48), suggesting that the standard O_2_ cell culture conditions suppress the actual oxidative capacity of cancer cells *in vitro*. Hypoxic conditions, on the other hand, have been shown to induce glycolysis (30–34, 49–51) and decrease the overall levels of TCA cycle metabolites and oxidative metabolism as compared to normoxia (33, 49, 50). Although diminished, mitochondrial-dependent metabolism did remain active at hypoxia (31, 33). For instance, Yang and colleagues (33) observed that exposing MDA-MB-231 breast cancer cells to hypoxia decreased the flux of glucose yet increased glutamine flux into the TCA cycle by enhancing glutaminolysis to compensate for the reduced mitochondrial metabolism under hypoxia. In addition to changes in energy metabolism, several studies report increased protein- (31, 33) and lipid catabolism (31, 48) under hypoxic conditions (1% O_2_). These increased catabolic reactions likely occur to compensate for the impaired mitochondrial activity that could not be corrected by increasing the glycolytic flux (31).

Subjecting cancer cells to physiologically relevant O_2_ levels might result in metabolic profiles that more accurately reflect tumor metabolism *in vivo* (48). For example, Blandin and colleagues demonstrated that the hypoxia-induced metabolic switch in cultured pediatric high-grade glioma (pHGG) cells was similar to the metabolic profile of matched relapsed pHGG tumors, indicating that culturing pHGG cells at 1% O_2_ more closely reflects patient tumor metabolism than cells cultured at normoxia (48). By subjecting identical PDAC surgical samples to 20% or 1% O_2_, Kumano et al. (52) found that hypoxia generated PDAC organoids with a different morphology, increased EMT-related protein expression and a higher 5-FU resistance. Their results suggest that hypoxia selects for PDAC cells with malignant traits, aiding in the development of effective anticancer treatments.

### 4.2 pH: neutral versus acidic

As with oxygen, culturing cancer cells at a tumor-like acidic pH reprograms their metabolism, revealing vulnerabilities that could improve the prediction of therapeutic effectiveness *in vivo*. Various studies compared the energy metabolism of cancer cells cultured at physiologic pH 7.4 or acidic pH ranging from 6.0 to 7.0. These revealed that acidosis reduced the general glucose uptake in tumor cells (35, 37, 54), and redirected glucose away from glycolysis and lactate production (35–40, 54) towards oxidative metabolism (36, 39, 53–56). Furthermore, several studies report an increased concomitant use of fatty acid (FA) breakdown and synthesis in cancer cells at acidic pH (37, 41). The latter process endows acid-adapted cancer cells with an increased capacity for utilizing FA for metabolic needs, while limiting glycolysis (41). Moreover, cells experiencing acidosis shift their metabolism towards the pentose phosphate pathway (PPP) (55, 56), important for the production of NADPH. NADPH is crucial for antioxidant defense, and acts in part by recycling glutathione (GSH) to counteract reactive oxygen species (ROS) (38, 105). Several papers report diminished GSH synthesis at low pH (38, 54), thereby increasing the demand for PPP-derived NADPH to recycle existing GSH pools (38). These findings indicating that low environmental pH affects both energy and redox metabolism to maintain homeostasis under acidosis-induced oxidative stress.

Acidosis-induced metabolic rewiring of cancer cells results in novel metabolic vulnerabilities that could potentially be exploited. As shown by Peppicelli et al. (36), treating acid-exposed melanoma cells with the mitochondrial complex I inhibitor Metformin inhibited the acidosis-induced oxidative metabolism and reduced the proliferation and epithelial-to-mesenchymal transition (EMT) of invasive melanoma cells. Chano et al. (55) showed a higher sensitivity of osteosarcoma cells to HDAC inhibitors at pH 6.5, suggesting that acidosis promotes metabolic profiles that contribute to epigenetic maintenance. Lastly, Corbet et al. (39) showed that targeting acid-driven glutamine metabolism *in vivo* with the glutaminase inhibitor BPTES significantly reduced the growth of tumors comprised of cells pre-adapted to pH 6.5, compared to tumors from cells adapted to neutral pH 7.4.

## 5 Discussion

Most of our current knowledge on cancer cell metabolism stems from reductionist *in vitro* models grown in a highly artificial environment. In this review, we present how current standard cell culture practices markedly influence cancer cell metabolism and how the use of more physiologically relevant culture conditions could mitigate discrepancies between *in vitro* and *in vivo* findings. The main findings of cited studies are compiled in **Table 1**. Summarized by us in **Figure 1** are the current recommendations for standard cell culture practices that could improve the metabolic fidelity of *in vitro* modeling systems.

However, not all factors described are easily implementable. For example, measuring metabolic activity under low O_2_ conditions requires the use of expensive hypoxic chambers and incubators that are not routinely used in most laboratories. Furthermore, not all cell culture models can currently be grown or maintained in a 3D setting. Finally, the development of the more complex CoC models requires interdisciplinary knowledge, ranging from biology to microfluidic chip engineering.

Still, tremendous progress has been made in optimizing the culture conditions and models to more accurately study cancer metabolism *in vitro*. Nevertheless, it is expected that our increasing knowledge on TME physiology as well as continuing technological advances will ultimately result in more representative models to study cancer metabolism. Presumably, these improvements will generate novel and meaningful insights into cancer metabolism that will be more effectively translated into successful anti-cancer therapies.

## References

[1] De Berardinis RJ Chandel NS. Fundamentals of cancer metabolism. Sci Adv. (2016) 2. doi: 10.1126/SCIADV.160020 PMC4928883 27386546

[2] Hanahan D Weinberg RA. Hallmarks of cancer: the next generation. Cell. (2011) 144:646–74. doi: 10.1016/J.CELL.2011.02.013 21376230

[3] Pavlova NN Thompson CB. The emerging hallmarks of cancer metabolism. Cell Metab. (2016) 23:27–47. doi: 10.1016/J.CMET.2015.12.006 26771115 PMC4715268

[4] Warburg O. On the origin of cancer cells. Science. (1956) 123:309–14. doi: 10.1126/SCIENCE.123.3191.309 13298683

[5] Wolpaw AJ Dang CV. Exploiting metabolic vulnerabilities of cancer with precision and accuracy. Trends Cell Biol. (2018) 28:201–12. doi: 10.1016/J.TCB.2017.11.006 PMC5818322 29229182

[6] Diamond LK Mercer RD Sylvester RF Wolff JA. Temporary remissions in acute leukemia in children produced by folic acid antagonist, 4-aminopteroyl-glutamic acid. N Engl J Med. (1948) 238:787–93. doi: 10.1056/NEJM194806032382301 18860765

[7] Danzi F Pacchiana R Mafficini A Scupoli MT Scarpa A Donadelli M. To metabolomics and beyond: a technological portfolio to investigate cancer metabolism. Signal Transduction Targeted Ther. (2023) 8:1–22. doi: 10.1038/s41392-023-01380-0 PMC10033890 36949046

[8] Lambie DG Johnson RH. Drugs and folate metabolism. Drugs. (1985) 30:145–55. doi: 10.2165/00003495-198530020-00003/METRICS 3896745

[9] Kaye SB. New antimetabolites in cancer chemotherapy and their clinical impact. Br J Cancer. (1998) 78:1. doi: 10.1038/BJC.1998.747 PMC2062805 9717984

[10] Dragic H Chaveroux C Cosset E Manie SN. Modelling cancer metabolism *in vitro*: current improvements and future challenges. FEBS J. (2024) 291:402–11. doi: 10.1111/FEBS.16704 36516350

[11] Davidson SM Papagiannakopoulos T Olenchock BA Heyman JE Keibler MA Luengo A. Environment impacts the metabolic dependencies of ras-driven non-small cell lung cancer. Cell Metab. (2016) 23:517–28. doi: 10.1016/J.CMET.2016.01.007 PMC4785096 26853747

[12] Cantor JR. The rise of physiologic media. Trends Cell Biol. (2019) 29:854. doi: 10.1016/J.TCB.2019.08.009 31623927 PMC7001851

[13] Golikov MV Valuev-Elliston VT Smirnova OA Ivanov AV. Physiological media in studies of cell metabolism. Mol Biol. (2022) 56:629–37. doi: 10.1134/S0026893322050077 PMC9534458 36217338

[14] Kapałczyńska M Kolenda T Przybyła W Zajączkowska M Teresiak A Filas V. 2D and 3D cell cultures – a comparison of different types of cancer cell cultures. Arch Med Sci. (2018) 14:910. doi: 10.5114/AOMS.2016.63743 30002710 PMC6040128

[15] Griffith LG Swartz MA. Capturing complex 3D tissue physiology *in vitro* . Nat Rev Mol Cell Biol. (2006) 7:211–24. doi: 10.1038/nrm1858 16496023

[16] Templeton AR Jeffery PL Thomas PB Perera MPJ Ng G Calabrese AR. Patient-derived explants as a precision medicine patient-proximal testing platform informing cancer management. Front Oncol. (2021) 11:767697. doi: 10.3389/FONC.2021.767697 34988013 PMC8721047

[17] Polak R Zhang ET Kuo CJ. Cancer organoids 2.0: modelling the complexity of the tumour immune microenvironment. Nat Rev Cancer. (2024) 24:523–39. doi: 10.1038/s41568-024-00706-6 38977835

[18] Pampaloni F Reynaud EG Stelzer EHK. The third dimension bridges the gap between cell culture and live tissue. Nat Rev Mol Cell Biol. (2007) 8:839–45. doi: 10.1038/nrm2236 17684528

[19] Rodríguez-Enríquez S Gallardo-Pérez JC Avilés-Salas A Marín-Hernández A Carreño-Fuentes L Maldonado-Lagunas V. Energy metabolism transition in multi-cellular human tumor spheroids. J Cell Physiol. (2008) 216:189–97. doi: 10.1002/JCP.21392 18264981

[20] Tidwell TR Røsland GV Tronstad KJ Søreide K Hagland HR. Metabolic flux analysis of 3D spheroids reveals significant differences in glucose metabolism from matched 2D cultures of colorectal cancer and pancreatic ductal adenocarcinoma cell lines. Cancer Metab. (2022) 10:1–16. doi: 10.1186/S40170-022-00285-W 35578327 PMC9109327

[21] Sato M Kawana K Adachi K Fujimoto A Yoshida M Nakamura H. Spheroid cancer stem cells display reprogrammed metabolism and obtain energy by actively running the tricarboxylic acid (TCA) cycle. Oncotarget. (2016) 7:33297. doi: 10.18632/ONCOTARGET.8947 27120812 PMC5078095

[22] Tobias F Hummon AB. Lipidomic comparison of 2D and 3D colon cancer cell culture models. J Mass Spectrom. (2022) 57(8):e4880. doi: 10.1002/JMS.4880 36028991 PMC9526240

[23] Vidavsky N Kunitake JAMR Diaz-Rubio ME Chiou AE Loh HC Zhang S. Mapping and profiling lipid distribution in a 3D model of breast cancer progression. ACS Cent Sci. (2019) 5:768–80. doi: 10.1021/ACSCENTSCI.8B00932 PMC6535773 31139713

[24] Fan TWM El-Amouri SS Macedo JKA Wang QJ Song H Cassel T. Stable isotope-resolved metabolomics shows metabolic resistance to anti-cancer selenite in 3D spheroids versus 2D cell cultures. Metabolites. (2018) 8(3):40. doi: 10.3390/METABO8030040 29996515 PMC6161115

[25] Russell S Wojtkowiak J Neilson A Gillies RJ. Metabolic Profiling of healthy and cancerous tissues in 2D and 3D. Sci Rep. (2017) 7:1–11. doi: 10.1038/s41598-017-15325-5 29127321 PMC5681543

[26] Cantor JR Abu-Remaileh M Kanarek N Freinkman E Gao X Louissaint A. Physiologic medium rewires cellular metabolism and reveals uric acid as an endogenous inhibitor of UMP synthase. Cell. (2017) 169:258–272.e17. doi: 10.1016/J.CELL.2017.03.023 28388410 PMC5421364

[27] Vande Voorde J Ackermann T Pfetzer N Sumpton D Mackay G Kalna G. Improving the metabolic fidelity of cancer models with a physiological cell culture medium. Sci Adv. (2019) 5(1):eaau7314. doi: 10.1126/SCIADV.AAU7314 30613774 PMC6314821

[28] Golikov MV Karpenko IL Lipatova AV Ivanova ON Fedyakina IT Larichev VF. Cultivation of cells in a physiological plasmax medium increases mitochondrial respiratory capacity and reduces replication levels of RNA viruses. Antioxidants (Basel). (2021) 11(1):97. doi: 10.3390/ANTIOX11010097 35052601 PMC8772912

[29] Moradi F Moffatt C Stuart JA. The effect of oxygen and micronutrient composition of cell growth media on cancer cell bioenergetics and mitochondrial networks. Biomolecules. (2021) 11(8):1177. doi: 10.3390/BIOM11081177 34439843 PMC8391631

[30] Timpano S Guild BD Specker EJ Melanson G Medeiros PJ Sproul SLJ. Physioxic human cell culture improves viability, metabolism, and mitochondrial morphology while reducing DNA damage. FASEB J. (2019) 33:5716–28. doi: 10.1096/FJ.201802279R 30649960

[31] Frezza C Zheng L Tennant DA Papkovsky DB Hedley BA Kalna G. Metabolic profiling of hypoxic cells revealed a catabolic signature required for cell survival. PloS One. (2011) 6:e24411. doi: 10.1371/JOURNAL.PONE.0024411 21912692 PMC3166325

[32] Tsai IL Kuo TC Ho TJ Harn YC Wang SY Fu WM. Metabolomic dynamic analysis of hypoxia in MDA-MB-231 and the comparison with inferred metabolites from transcriptomics data. Cancers (Basel). (2013) 5:491. doi: 10.3390/CANCERS5020491 24216987 PMC3730319

[33] Yang J Cheng J Sun B Li H Wu S Dong F. Untargeted and stable isotope-assisted metabolomic analysis of MDA-MB-231 cells under hypoxia. Metabolomics. (2018) 14:1–14. doi: 10.1007/S11306-018-1338-8 30830323

[34] Martín-Bernabé A Tarragó-Celada J Cunin V Michelland S Cortés R Poignant J. Quantitative proteomic approach reveals altered metabolic pathways in response to the inhibition of lysine deacetylases in A549 cells under normoxia and hypoxia. Int J Mol Sci. (2021) 22(7):3378. doi: 10.3390/IJMS22073378 33806075 PMC8036653

[35] Chen JLY Lucas JE Schroeder T Mori S Wu J Nevins J. The genomic analysis of lactic acidosis and acidosis response in human cancers. PloS Genet. (2008) 4(12):e1000293. doi: 10.1371/JOURNAL.PGEN.1000293 19057672 PMC2585811

[36] Peppicelli S Toti A Giannoni E Bianchini F Margheri F Del Rosso M. Metformin is also effective on lactic acidosis-exposed melanoma cells switched to oxidative phosphorylation. Cell Cycle. (2016) 15:1908. doi: 10.1080/15384101.2016.1191706 27266957 PMC4968910

[37] Corbet C Pinto A Martherus R Santiago de Jesus JP Polet F Feron O. Acidosis drives the reprogramming of fatty acid metabolism in cancer cells through changes in mitochondrial and histone acetylation. Cell Metab. (2016) 24:311–23. doi: 10.1016/J.CMET.2016.07.003 27508876

[38] LaMonte G Tang X Chen JL-Y Wu J Ding C-KC Keenan MM. Acidosis induces reprogramming of cellular metabolism to mitigate oxidative stress. Cancer Metab. (2013) 1:23. doi: 10.1186/2049-3002-1-23 24359630 PMC4178214

[39] Corbet C Draoui N Polet F Pinto A Drozak X Riant O. The SIRT1/HIF2α axis drives reductive glutamine metabolism under chronic acidosis and alters tumor response to therapy. Cancer Res. (2014) 74:5507–19. doi: 10.1158/0008-5472.CAN-14-0705 25085245

[40] Prado-Garcia H Campa-Higareda A Romero-Garcia S. Lactic acidosis in the presence of glucose diminishes warburg effect in lung adenocarcinoma cells. Front Oncol. (2020) 10:807/BIBTEX. doi: 10.3389/FONC.2020.00807/BIBTEX 32596143 PMC7303336

[41] Rolver MG Holland LKK Ponniah M Prasad NS Yao J Schnipper J. Chronic acidosis rewires cancer cell metabolism through PPARα signaling. Int J Cancer. (2023) 152:1668–84. doi: 10.1002/IJC.34404 PMC10108231 36533672

[42] Ikari R Mukaisho KI Kageyama S Nagasawa M Kubota S Nakayama T. Differences in the central energy metabolism of cancer cells between conventional 2D and novel 3D culture systems. Int J Mol Sci. (2021) 22:1–13. doi: 10.3390/IJMS22041805 PMC7917672 33670390

[43] Wen S Tu X Zang Q Zhu Y Li L Zhang R. Liquid chromatography–mass spectrometry-based metabolomics and fluxomics reveals the metabolic alterations in glioma U87MG multicellular tumor spheroids versus two-dimensional cell cultures. Rapid Commun Mass Spectrometry. (2024) 38:e9670. doi: 10.1002/RCM.9670 38124173

[44] Murakami S Tanaka H Nakayama T Taniura N Miyake T Tani M. Similarities and differences in metabolites of tongue cancer cells among two- and three-dimensional cultures and xenografts. Cancer Sci. (2021) 112:918–31. doi: 10.1111/CAS.14749 PMC7894009 33244783

[45] Zang Q Sun C Chu X Li L Gan W Zhao Z. Spatially resolved metabolomics combined with multicellular tumor spheroids to discover cancer tissue relevant metabolic signatures. Anal Chim Acta. (2021) 1155:338342. doi: 10.1016/J.ACA.2021.338342 33766316

[46] Saab JJA Dzierozynski LN Jonker PB Aminitabrizi R Shah H Menjivar RE. Pancreatic tumors exhibit myeloid-driven amino acid stress and upregulate arginine biosynthesis. Elife. (2023) 12:e81289. doi: 10.7554/ELIFE.81289 37254839 PMC10260022

[47] Khadka S Arthur K Barekatain Y Behr E Washington M Ackroyd J. Impaired anaplerosis is a major contributor to glycolysis inhibitor toxicity in glioma. Cancer Metab. (2021) 9(1):27. doi: 10.1186/S40170-021-00259-4 34172075 PMC8228515

[48] Blandin AF Durand A Litzler M Tripp A Guérin É Ruhland E. Hypoxic environment and paired hierarchical 3D and 2D models of pediatric H3.3-mutated gliomas recreate the patient tumor complexity. Cancers (Basel). (2019) 11(12):1875. doi: 10.3390/CANCERS11121875 31779235 PMC6966513

[49] Gunda V Kumar S Dasgupta A Singh PK. Hypoxia-induced metabolomic alterations in pancreatic cancer cells. Methods Mol Biol. (2018) 1742:95–105. doi: 10.1007/978-1-4939-7665-2_9 29330793

[50] Kucharzewska P Christianson HC Belting M. Global profiling of metabolic adaptation to hypoxic stress in human glioblastoma cells. PloS One. (2015) 10(1):e0116740. doi: 10.1371/JOURNAL.PONE.0116740 25633823 PMC4310608

[51] Al-Mutawa YK Herrmann A Corbishley C Losty PD Phelan M Sée V. Effects of hypoxic preconditioning on neuroblastoma tumour oxygenation and metabolic signature in a chick embryo model. Biosci Rep. (2018) 38:20180185. doi: 10.1042/BSR20180185 PMC6131206 30026261

[52] Kumano K Nakahashi H Louphrasitthiphol P Kuroda Y Miyazaki Y Shimomura O. Hypoxia at 3D organoid establishment selects essential subclones within heterogenous pancreatic cancer. Front Cell Dev Biol. (2024) 12:1327772. 38374892 10.3389/fcell.2024.1327772 PMC10875002

[53] Degitz C Reime S Baumbach CM Rauschner M Thews O. Modulation of mitochondrial function by extracellular acidosis in tumor cells and normal fibroblasts: Role of signaling pathways. Neoplasia. (2019) 52:100999. doi: 10.1016/j.neo.2024.100999 PMC11036092 38631214

[54] Abrego J Gunda V Vernucci E Shukla SK King RJ Dasgupta A. GOT1-mediated anaplerotic glutamine metabolism regulates chronic acidosis stress in pancreatic cancer cells. Cancer Lett. (2017) 400:37. doi: 10.1016/J.CANLET.2017.04.029 28455244 PMC5488721

[55] Chano T Avnet S Kusuzaki K Bonuccelli G Sonveaux P Rotili D. Tumour-specific metabolic adaptation to acidosis is coupled to epigenetic stability in osteosarcoma cells. Am J Cancer Res. (2016) 6:859. 27186436 PMC4859889

[56] Xu X Wang L Zang Q Li S Li L Wang Z. Rewiring of purine metabolism in response to acidosis stress in glioma stem cells. Cell Death Dis. (2021) 12(3):277. doi: 10.1038/S41419-021-03543-9 33723244 PMC7961141

[57] Lovitt CJ Shelper TB Avery VM. Evaluation of chemotherapeutics in a three-dimensional breast cancer model. J Cancer Res Clin Oncol. (2015) 141:951–9. doi: 10.1007/S00432-015-1950-1 PMC11823512 25773123

[58] Longati P Jia X Eimer J Wagman A Witt MR Rehnmark S. 3D pancreatic carcinoma spheroids induce a matrix-rich, chemoresistant phenotype offering a better model for drug testing. BMC Cancer. (2013) 13:1–13. doi: 10.1186/1471-2407-13-95 23446043 PMC3617005

[59] Breslin S O’Driscoll L. The relevance of using 3D cell cultures, in addition to 2D monolayer cultures, when evaluating breast cancer drug sensitivity and resistance. Oncotarget. (2016) 7:45745. doi: 10.18632/ONCOTARGET.9935 27304190 PMC5216757

[60] Maddocks ODK Athineos D Cheung EC Lee P Zhang T Van Den Broek NJF. Modulating the therapeutic response of tumours to dietary serine and glycine starvation. Nature. (2017) 544:372–6. doi: 10.1038/nature22056 28425994

[61] Yoshizaki H Ogiso H Okazaki T Kiyokawa E. Comparative lipid analysis in the normal and cancerous organoids of MDCK cells. J Biochem. (2016) 159:573–84. doi: 10.1093/JB/MVW001 PMC4892391 26783265

[62] Lindeboom RG van Voorthuijsen L Oost KC Rodríguez-Colman MJ Luna-Velez MV Furlan C. Integrative multi-omics analysis of intestinal organoid differentiation. Mol Syst Biol. (2018) 14:8227. doi: 10.15252/MSB.20188227 PMC6018986 29945941

[63] Neef SK Janssen N Winter S Wallisch SK Hofmann U Dahlke MH. Metabolic drug response phenotyping in colorectal cancer organoids by LC-QTOF-MS. Metabolites. (2020) 10:494. doi: 10.3390/METABO10120494 33271860 PMC7760698

[64] Ludikhuize MC Gevers S Nguyen NTB Meerlo M Roudbari SKS Gulersonmez MC. Rewiring glucose metabolism improves 5-FU efficacy in p53-deficient/KRASG12D glycolytic colorectal tumors. Commun Biol. (2022) 5(1):1159. doi: 10.1038/S42003-022-04055-8 36316440 PMC9622833

[65] Rebeaud M Bouche C Dauvillier S Attané C Arellano C Vaysse C. A novel 3D culture model for human primary mammary adipocytes to study their metabolic crosstalk with breast cancer in lean and obese conditions. Sci Rep. (2023) 13:1–12. doi: 10.1038/s41598-023-31673-x 36949082 PMC10033714

[66] Wang YY Attané C Milhas D Dirat B Dauvillier S Guerard A. Mammary adipocytes stimulate breast cancer invasion through metabolic remodeling of tumor cells. JCI Insight. (2017) 2. doi: 10.1172/JCI.INSIGHT.87489 PMC5313068 28239646

[67] Olszańska J Pietraszek-Gremplewicz K Domagalski M Nowak D. Mutual impact of adipocytes and colorectal cancer cells growing in co-culture conditions. Cell Communication Signaling. (2023) 21:1–18. doi: 10.1186/S12964-023-01155-8 37316878 PMC10265888

[68] Asante EC Pallegar NK Viloria-Petit AM Christian SL. Three-dimensional co-culture method for studying interactions between adipocytes, extracellular matrix, and cancer cells. Methods Mol Biol. (2022) 2508:69–77. doi: 10.1007/978-1-0716-2376-3_7 35737234

[69] Acevedo-Acevedo S Millar DC Simmons AD Favreau P Cobra PF Skala M. Metabolomics revealed the influence of breast cancer on lymphatic endothelial cell metabolism, metabolic crosstalk, and lymphangiogenic signaling in co-culture. Sci Rep. (2020) 10(1):21244. doi: 10.1038/S41598-020-76394-7 33277521 PMC7718899

[70] Halama A Guerrouahen BS Pasquier J Satheesh NJ Suhre K Rafii A. Nesting of colon and ovarian cancer cells in the endothelial niche is associated with alterations in glycan and lipid metabolism. Sci Rep. (2017) 7:1–10. doi: 10.1038/srep39999 28051182 PMC5209689

[71] Strating E Verhagen MP Wensink E Dünnebach E Wijler L Aranguren I. Co-cultures of colon cancer cells and cancer-associated fibroblasts recapitulate the aggressive features of mesenchymal-like colon cancer. Front Immunol. (2023) 14:1053920/BIBTEX. doi: 10.3389/FIMMU.2023.1053920 37261365 PMC10228738

[72] Koukourakis MI Kalamida D Mitrakas AG Liousia M Pouliliou S Sivridis E. Metabolic cooperation between co-cultured lung cancer cells and lung fibroblasts. Lab Invest. (2017) 97:1321–31. doi: 10.1038/labinvest.2017.79 28846077

[73] Goossens P Rodriguez-Vita J Etzerodt A Masse M Rastoin O Gouirand V. Membrane cholesterol efflux drives tumor-associated macrophage reprogramming and tumor progression. Cell Metab. (2019) 29:1376–1389.e4. doi: 10.1016/J.CMET.2019.02.016 30930171

[74] Yang P Qin H Li Y Xiao A Zheng E Zeng H. CD36-mediated metabolic crosstalk between tumor cells and macrophages affects liver metastasis. Nat Commun. (2022) 13:1–16. doi: 10.1038/s41467-022-33349-y 36184646 PMC9527239

[75] Raffo-Romero A Ziane-Chaouche L Salomé-Desnoulez S Hajjaji N Fournier I Salzet M. A co-culture system of macrophages with breast cancer tumoroids to study cell interactions and therapeutic responses. Cell Rep Methods. (2024) 4(6):100792. doi: 10.1016/j.crmeth.2024.100792 38861990 PMC11228374

[76] Chen P Han Y Wang L Zheng Y Zhu Z Zhao Y. Spatially resolved metabolomics combined with the 3D tumor-immune cell coculture spheroid highlights metabolic alterations during antitumor immune response. Anal Chem. (2023) 95:15153–61. doi: 10.1021/ACS.ANALCHEM.2C05734 37800909

[77] Jeong SR Kang M. Exploring tumor–immune interactions in co-culture models of T cells and tumor organoids derived from patients. Int J Mol Sci. (2023) 24:14609. doi: 10.3390/IJMS241914609 37834057 PMC10572813

[78] Bader JE Voss K Rathmell JC. Targeting metabolism to improve the tumor microenvironment for cancer immunotherapy. Mol Cell. (2020) 78:1019. doi: 10.1016/J.MOLCEL.2020.05.034 32559423 PMC7339967

[79] Kenerson HL Sullivan KM Seo YD Stadeli KM Ussakli C Yan X. Tumor slice culture as a biologic surrogate of human cancer. Ann Transl Med. (2020) 8:114–4. doi: 10.21037/ATM.2019.12.88 PMC7049013 32175407

[80] Mendes R Graça G Silva F Guerreiro ACL Gomes-Alves P Serpa J. Exploring metabolic signatures of ex vivo tumor tissue cultures for prediction of chemosensitivity in ovarian cancer. Cancers (Basel). (2022) 14(18):4460. doi: 10.3390/CANCERS14184460 36139619 PMC9496731

[81] Sellers K Fox MP Ii MB Slone SP Higashi RM Miller DM. Pyruvate carboxylase is critical for non-small-cell lung cancer proliferation. J Clin Invest. (2015) 125:687–98. doi: 10.1172/JCI72873 PMC4319441 25607840

[82] Ko J Park D Lee S Gumuscu B Jeon NL. Engineering organ-on-a-chip to accelerate translational research. Micromachines. (2022) 13:1200. doi: 10.3390/MI13081200 36014122 PMC9412404

[83] Mastrangeli M Millet S Van den Eijnden-van Raaij J. Organ-on-chip in development: Towards a roadmap for organs-on-chip. ALTEX. (2019) 36:650–68. doi: 10.14573/ALTEX.1908271 31664458

[84] Lopez-Muñoz GA Mugal S Ramón-Azcón J. Sensors and biosensors in organs-on-a-chip platforms. Adv Exp Med Biol. (2022) 1379:55–80. doi: 10.1007/978-3-031-04039-9_3 35760988

[85] Dornhof J Kieninger J Muralidharan H Maurer J Urban GA Weltin A. Microfluidic organ-on-chip system for multi-analyte monitoring of metabolites in 3D cell cultures. Lab Chip. (2022) 22:225–39. doi: 10.1039/D1LC00689D 34851349

[86] Kalfe A Telfah A Lambert J Hergenröder R. Looking into living cell systems: planar waveguide microfluidic NMR detector for *in vitro* metabolomics of tumor spheroids. Anal Chem. (2015) 87:7402–10. doi: 10.1021/ACS.ANALCHEM.5B01603 26121119

[87] Chen Q Wu J Zhang Y Lin JM. Qualitative and quantitative analysis of tumor cell metabolism via stable isotope labeling assisted microfluidic chip electrospray ionization mass spectrometry. Anal Chem. (2012) 84:1695–701. doi: 10.1021/AC300003K 22242916

[88] Jeibouei S Hojat A Mostafavi E Aref AR Kalbasi A Niazi V. Radiobiological effects of wound fluid on breast cancer cell lines and human-derived tumor spheroids in 2D and microfluidic culture. Sci Rep. (2022) 12:1–21. doi: 10.1038/s41598-022-11023-z 35538133 PMC9091274

[89] Cauli E Polidoro MA Marzorati S Bernardi C Rasponi M Lleo A. Cancer-on-chip: a 3D model for the study of the tumor microenvironment. J Biol Eng. (2023) 17:1–25. doi: 10.1186/S13036-023-00372-6 37592292 PMC10436436

[90] Lobel GP Jiang Y Simon MC. Tumor microenvironmental nutrients, cellular responses, and cancer. Cell Chem Biol. (2023) 30:1015–32. doi: 10.1016/J.CHEMBIOL.2023.08.011 PMC10528750 37703882

[91] Gkiouli M Biechl P Eisenreich W Otto AM. Diverse roads taken by 13C-glucose-derived metabolites in breast cancer cells exposed to limiting glucose and glutamine conditions. Cells. (2019) 8(10):1113. doi: 10.3390/CELLS8101113 31547005 PMC6829299

[92] Nestor CE Ottaviano R Reinhardt D Cruickshanks HA Mjoseng HK McPherson RC. Rapid reprogramming of epigenetic and transcriptional profiles in mammalian culture systems. Genome Biol. (2015) 16:1–17. doi: 10.1186/S13059-014-0576-Y 25648825 PMC4334405

[93] Edgar RD Perrone F Foster AR Payne F Lewis S Nayak KM. Culture-associated DNA methylation changes impact on cellular function of human intestinal organoids. Cell Mol Gastroenterol Hepatol. (2022) 14:1295. doi: 10.1016/J.JCMGH.2022.08.008 36038072 PMC9703134

[94] Wiig H Swartz MA. Interstitial fluid and lymph formation and transport: Physiological regulation and roles in inflammation and cancer. Physiol Rev. (2012) 92:1005–60. doi: 10.1152/PHYSREV.00037.2011 22811424

[95] Sullivan MR Danai LV Lewis CA Chan SH Gui DY Kunchok T. Quantification of microenvironmental metabolites in murine cancers reveals determinants of tumor nutrient availability. Elife. (2019) 8:e44235. doi: 10.7554/ELIFE.44235 30990168 PMC6510537

[96] Gardner GL Moradi F Moffatt C Cliche M Garlisi B Gratton J. Rapid nutrient depletion to below the physiological range by cancer cells cultured in Plasmax. Am J Physiol Cell Physiol. (2022) 323:C823–34. doi: 10.1152/AJPCELL.00403.2021 35876286

[97] Hopkins E Sanvictores T Sharma S. Urolithiasis.StatPearls Publishing (Boston, MA: Springer) (2022). 19–22 p. doi: 10.1007/978-1-4899-0873-5_4

[98] Segeritz CP Vallier L. Cell culture: growing cells as model systems *in vitro* . In: Basic Science Methods for Clinical Researchers (2017). (Academic Press) p. 151. doi: 10.1016/B978-0-12-803077-6.00009-6

[99] Alva R Gardner GL Liang P Stuart JA. Supraphysiological oxygen levels in mammalian cell culture: current state and future perspectives. Cells. (2022) 11:3123. doi: 10.3390/CELLS11193123 36231085 PMC9563760

[100] McKeown SR. Defining normoxia, physoxia and hypoxia in tumours-implications for treatment response. Br J Radiol. (2014) 87(1035):20130676. doi: 10.1259/BJR.20130676 24588669 PMC4064601

[101] Kato Y Ozawa S Miyamoto C Maehata Y Suzuki A Maeda T. Acidic extracellular microenvironment and cancer. Cancer Cell Int. (2013) 13:89. doi: 10.1186/1475-2867-13-89 24004445 PMC3849184

[102] Vaupel P. Tumor microenvironmental physiology and its implications for radiation oncology. Semin Radiat Oncol. (2004) 14:198–206. doi: 10.1016/J.SEMRADONC.2004.04.008 15254862

[103] Hao G Xu ZP Li L. Manipulating extracellular tumour pH: an effective target for cancer therapy. RSC Adv. (2018) 8:22182–92. doi: 10.1039/C8RA02095G PMC9081285 35541713

[104] Abbas M Moradi F Hu W Regudo KL Osborne M Pettipas J. Vertebrate cell culture as an experimental approach - limitations and solutions. Comp Biochem Physiol B Biochem Mol Biol. (2021) 254:110570. doi: 10.1016/J.CBPB.2021.110570 33516822

[105] Mullarky E Cantley LC. Diverting glycolysis to combat oxidative stress. Innovative Med. (2015), 3–23. doi: 10.1007/978-4-431-55651-0_1 29787184

